# Suppression of human papillomavirus type 16 E5 oncoprotein: A promising step in fostering the treatment of cervical cancer

**DOI:** 10.32604/or.2022.023346

**Published:** 2022-07-13

**Authors:** NIMA HEMMAT, MOHAMMAD AMIN DOUSTVANDI, ZAHRA ASADZADEH, AHAD MOKHTARZADEH, BEHZAD BARADARAN, HOSSEIN BANNAZADEH BAGHI

**Affiliations:** 1 Immunology Research Center, Tabriz University of Medical Sciences, Tabriz, Iran; 2 Infectious and Tropical Diseases Research Center, Tabriz University of Medical Sciences, Tabriz, Iran; 3 Department of Virology, Faculty of Medicine, Tabriz University of Medical Sciences, Tabriz, Iran; 4 Department of Immunology, Faculty of Medicine, Tabriz University of Medical Sciences, Tabriz, Iran

**Keywords:** Human papillomavirus, e5 transforming protein, epidermal growth factor receptor, cervical cancer, CaSki

## Abstract

Cervical cancer is a growing global disease in developing countries. Persistent infection with human papillomaviruses (HPV) is an essential causative agent in this type of cancer. Several studies demonstrate HPV E5 oncoprotein can impress the normal life cycle of HPV-infected cells by targeting some pivotal cellular signaling pathways, such as the epidermal growth factor receptor (EGFR) signaling pathway. In this study, we used E5-siRNA to knockdown that essential oncogene and considered the effect of E5 silencing on proliferation, apoptosis, cell cycle, apoptosis-related gene expression, and the initiator of the EGFR signaling pathway in cervical cancer cells. The results demonstrate that E5 plays an essential role in the proliferation and inhibited apoptosis in cervical cancer. Furthermore, silencing E5 reduces proliferation, increases apoptosis, and elevates related-genes expression of these malignant cells. Overall, E5 suppression may be appropriate for ameliorating cervical cancer progression.

## Introduction

Cervical cancer is a growing global disease in developing countries. Approximately 528,000 new cancer cases and a mortality of 266,000 were attributed to this gynecologic cancer in 2012. Also, it is reported that approximately 604,000 new cases of women were diagnosed with cervical cancer around the world, and around 342,000 women died from it in 2020 [1]. Internationally, cervical cancer is the fourth most rampant cancer in women. Several risk factors could contribute to the induction of cervical cancer, such as smoking or HIV infection; however, persistent infection with human papillomaviruses (HPV) is an essential causative agent in this type of cancer [2]. HPV-16 and -18 were blamed for 75% of cervical cancer cases worldwide. Another 10% of these cancers are due to type 31 and 45 infections [3]. Regarding the early or late expression in the epithelial cells and transforming process, HPV genes are classified into Early proteins (E) and Late proteins (L) [4].

The E5 proteins are encoded by the HPV, which was previously supposed to have weak transforming activity. However, several recent studies have demonstrated that this oncoprotein can also impress the normal life cycle of HPV-infected cells [5]. HPV E5 protein could be noticed as an oncogene functioning at the early stage of oncogenesis [6]. This protein’s localization to the endoplasmic reticulum indicates that its function can be related to the traffic in cytoplasmic membrane proteins, particularly in host immune control and related receptors and molecules. Several studies demonstrate intracellular binding targets for E5, including some members of the epidermal growth factor receptor (EGFR) family [7,8]. EGFR overactivation by E5 also interacts with the inflammatory pathway. Following phosphorylation of EGFR, this receptor is able to stimulate cyclooxygenase 2 (COX-2 or PTGS2), which is usually associated with malignancies and a decreased overall survival rate and high metastasis [9,10].

Despite the common therapeutic approaches employed for cancer treatment, targeted therapy is recommended today to minimize side effects and improve treatment performance. A specific type of targeted therapy is gene therapy, which uses molecular methods to treat diseases that are caused by genetic alterations. In recent years, gene therapy has been particularly prominent in the mechanism of treatment [11]. The RNA interference (RNAi) technique is a process in which a double-stranded RNA is inserted into the cell cytoplasm, which cuts and destroys the target gene’s mRNAs, thereby suppressing gene expression [12]. These short interfering RNAs, which are 21–23 nucleotides in length and processed by nucleases associated with RNA polymerase III, are called small interfering RNA (siRNA) [13].

The importance of HPV oncogenes as the target of therapeutic methods for HPV-related cancers is highlighted through their expression exclusively in malignant cells. Therefore, methods by which the expression and activity of HPV oncoprotein are affected can stand in the first-line treatment of such cancer. Initially, Jiang et al. utilized siRNA to reduce the expression of HPV E6 and E7 oncogenes to ameliorate the oncogenesis of the cervical cancer cells. Their data demonstrated that the silencing of E6 and E7 expression gives rise to the accumulation of p53 in transfected cells and an increased apoptosis rate [14]. The silencing of the HPV E5 oncogene was firstly exerted by Oh et al. in 2009 [15]. Their study reported that the transfection of E5-positive cancer cells with HPV E5 siRNA could decrease the expression of E5 targets in transfected cells such as PTGS2. To complete these previously published results, in this experimental study, we used siRNA with the ability to silence HPV-16 E5 oncogene (E5-siRNA) to find the effect of E5 suppression on the several aspects of the cellular process which are essential in the maintenance of cancerous status in cervical cancer cells such as the apoptosis, cell cycle, and their related genes. The results of current study could be beneficial in designing a therapeutic approach based on the inhibition of HPV oncogenes functions.

## Materials and Methods

### Cell culture

CaSki cells, the human cervical cancer cells containing integrated HPV-16 genome and E5 transcripts, were obtained from the Iranian Biological Resource Center (IBRC). They were cultivated in the complete RPMI-1640 medium (Gibco, USA) supplemented with 10% Fetal Bovine Serum (FBS) (Gibco), as well as antibiotics including streptomycin (100 μg/mL) and penicillin (100 IU/mL), all gathered in T25 cell culture flask. The cultivation process was made in an incubator with 5% CO_2_ and 95% humidity conditions. The sub-culture procedure was done utilizing 0.25% Trypsin-EDTA (Gibco) to detach cells as they reached 70% confluence.

### siRNA transfection

Regarding the presence of the HPV-16 genome within the CaSki cells and the expression of E5 oncogene in these cells, this type of cervical cancer cell line was chosen for the following tests. E5-siRNA (Bioneer, South Korea) (Table 1) and FITC-labeled siRNA (scrambled siRNA) (GenePharma Co., Shanghai, China) were transfected within the CaSki cells with the serial doses using the Gene Pulser electroporation system (Bio-Rad). The scrambled siRNA was checked by nucleotide BLAST to find if there is no target sequence for this siRNA in the human transcriptome. Also, utilizing Nucleotide BLAST, the specificity of the designated siRNA was evaluated. The results showed that this siRNA could effectively target all the strains of HPV-16 and all transcript variants of E5. Following the application of the electroporation protocol (TC = 12.5 ms and Voltage = 160 v), the proper number of cells were seeded in different cell culture plates regarding the ongoing test. Also, the scrambled siRNA was transfected to the CaSki cells to assess the efficacy of transfection.

**Table 1:** The sequences of the sense and anti-sense strands of E5-siRNA

E5-siRNA	Sense strand	Anti-sense strand
	UUAAAAAGCGUGCAUGUGUdTdT	ACACAUGCACGCUUUUUAAdTdT

### qRT-PCR for gene expression analysis

GeneAll Trizol RNA extraction reagent (Korea) was utilized to extract the total RNA of each group. The absorbance measurement quantified the purity and concentration of isolated RNA at 260 and 280 nm wavelength employing the NanoDrop spectrophotometer (Thermo Fisher Scientific Life Sciences, USA). To measure the desired expression levels, cDNA synthesis was done using BioFACT^TM^ 2 Step 2X RT-PCR Pre-Mix (South Korea) at the 1000 ng/μl concentration of total RNA. Afterwards, the expression of HPV-16 E5, BCL2, BAX, EGFR, and COX-2 was determined using the BioFACT^TM^ Real-Time PCR Master Mix (South Korea) quantified by the StepOnePlus Real-Time PCR System (Applied Biosystems, USA). GAPDH was used as the internal control to normalize the expressions and performance of ΔΔCt analysis. In Table 2, the relevant sequences of the primer pairs are shown.

**Table 2:** The sequences of primer pairs used for qRT-PCR

Gene name	Type	Sequence (5’→ 3’)
E5	F	CCACAACATTACTGGCGTGC
	R	GCAGAGGCTGCTGTTATCCAC
EGFR	F	GGTGCAGGAGAGGAGAACTG
	R	GGCTTCGTCTCGGAATTTGC
COX-2 (PTGS2)	F	GCCTGAATGTGCCATAAGACTG
	R	CCACAGTGCTTGACACAGAA
BAX	F	TTTGCTTCAGGGTTTCATCCA
	R	CTCCATGTTACTGTCCAGTTCGT
BCL2	F	GAGCGTCAACAGGGAGATGTC
	R	TGCCGGTTCAGGTACTCAGTC
GAPDH	F	CAAGATCATCAGCAATGCCT
	R	GCCATCACGCCACAGTTTCC

### Determination of E5-siRNA cytotoxicity

3-(4, 5-dimethylthiazol-2-yl)-2, 5-diphenyltetrazolium bromide (MTT) assay was used to test CaSki cells’ cell viability in 24, 48, and 72 h after the transfection, as well as the viability of cells transfected with scrambled siRNA. Regarding the doubling time of CaSki cells (3.2 days), about 1.5 × 10^4^, 1.2 × 10^4^, and 1 × 10^4^ cells were seeded in 96-well for 24, 48, and 72 h groups, respectively. Following the mentioned incubation time, the cells within each well were treated with 50 μl of MTT solution (2 mg/ml) and incubated for 4 h. To prepare the formed formazan crystals for measuring the absorbance, the MTT solution was discarded from each well, and 150 μl of dimethyl sulfoxide (DMSO) was added. Then, employing the microplate reader (Tecan, Switzerland), the absorbance of each well was measured at 570 nm wavelength and 620 nm as the reference wavelength. All experiments were carried out triplicated.

### Wound healing (scratch) assay

The wound-healing assay was performed to find the effect of E5-siRNA on the migration ability of cervical cancer cells. The proper number of cells were transfected with E5-siRNA and then seeded in 24-well plates and the control groups. A straight and suitable width line was scratched after the adhesion of cells within the wells. Utilizing an inverted microscope (Optika, XDS-3, Italy), E5-siRNA-transfected cells and control cells’ migration rate were followed at 0, 12, 24, and 48 h.

### Apoptosis assay

The CaSki cells were transfected with E5-siRNA and then cultured within 6-well plates with a cell rate of 5 × 10^5^ per well. In addition, the same number of control cells (non-transfected cells) and scrambled siRNA-transfected cells were cultured on the plate. After 72 h following the transfection (optimized before) with 100 pmol of siRNA, all the groups were harvested, and the apoptosis rate was studied using the Annexin-V/PI staining kit (Exbio-Czech).

### Cell cycle assay

Following the transfection of cervical cancer cells (CaSki) with E5-siRNA, a 6-well plate was utilized to culture the transfected cells as well as control (non-transfected) cells and scrambled siRNA-transfected cells. After the appropriate time had elapsed (72 h), the cells were trypsinized and harvested, followed by the fixation with 70% ethanol for the overnight duration. Henceforth, all the cells in each group were stained with propidium iodide (PI). Proceeding to this step, all the cells should be treated with RNase A (Bioneer, Daejeon, Korea).

### Statistical analysis

Using GraphPad Prism v8 (San Diego, California, USA, www.graphpad.com), the significance of data was evaluated via student *t*-test and ANOVA one way and two ways. First, all the values were presented as mean ± SD, and the *p*-value < 0.05 was considered the significance cut-off criterion.

## Results

### Optimization of time-dependent and dose-dependent reduced expression of E5

**Figure 1:**
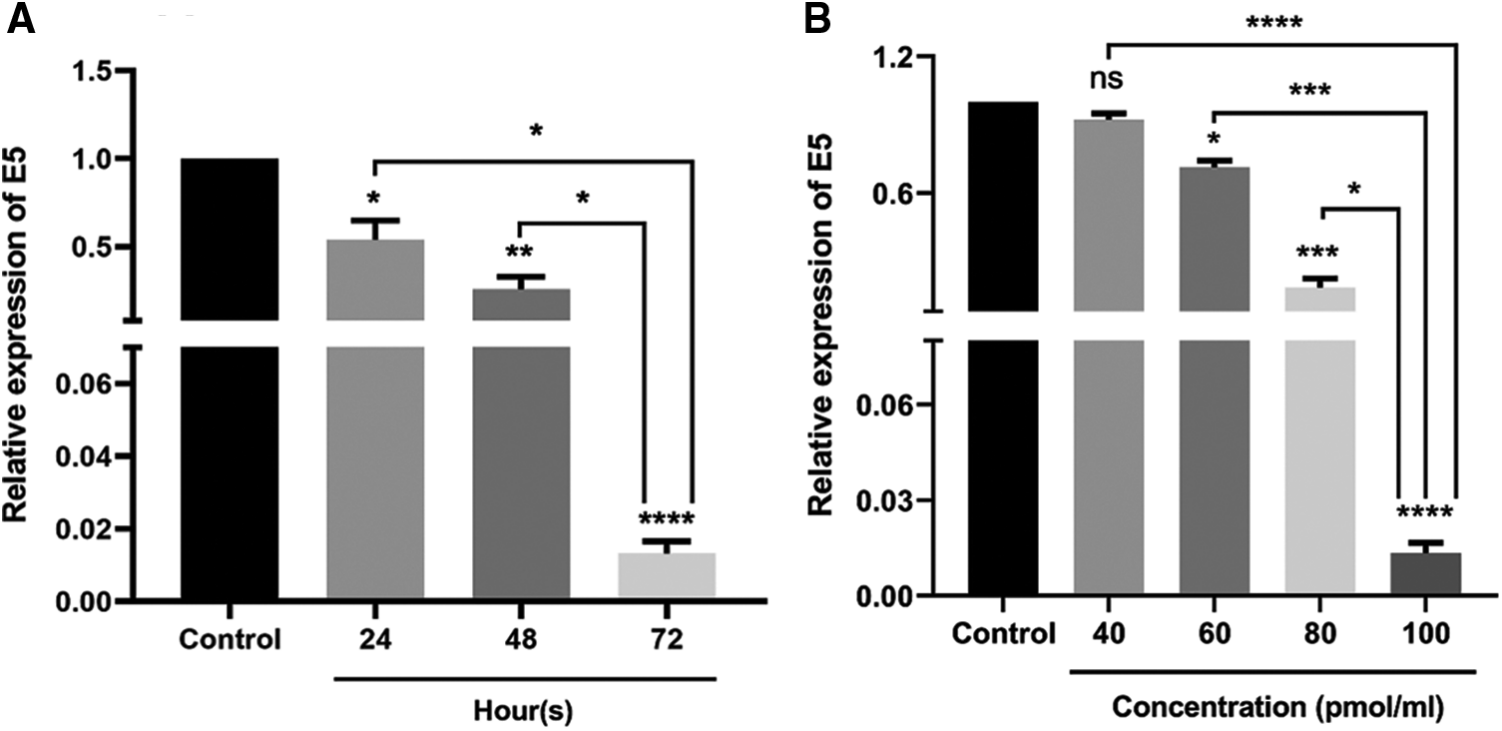
**A.** Effect of E5-siRNA on the expression of E5 at different times. The results showed that 72 h after the transfection is the best time for the optimum activity of E5-siRNA. **B.** Effect of E5-siRNA on the expression of E5 in different doses. 100 pmol of this siRNA could significantly reduce the expression of E5 in comparison with the control group and other tested doses (*p < 0.05, **p < 0.01, ***p < 0.001, ****p < 0.0001, n = 3.

The effect of E5-siRNA to reduce the expression of E5 at 24, 48, and 72 h after the transfection was evaluated by qRT-PCR. Also, several doses of E5-siRNA, including 40, 60, 80, and 100 pmol, were transfected to the cells to assess the efficacy of this siRNA on the expression of E5 in the mentioned doses. The results showed that this siRNA reduced E5 expression levels in a time-and dose-dependent manner compared with the control group. At 24, 48, and 72 h after the transfection of E5-siRNA, the decreased level of E5 expression was 46.08%, 76.3%, and 98.67%, respectively, compared to the control group (Fig. 1A). Moreover, 100 pmol of E5-siRNA had the best efficacy among the tested doses, as reported by the manufacturer (Fig. 1B). Therefore, in each repeat, the expression of E5 in the control group was assumed as 100% or 1. As mentioned, the significant decrease in the expression of E5 was reported to be after 72 h of the transfection.

### The decreased expression level of E5 could affect the survival rate of CaSki cells

**Figure 2:**
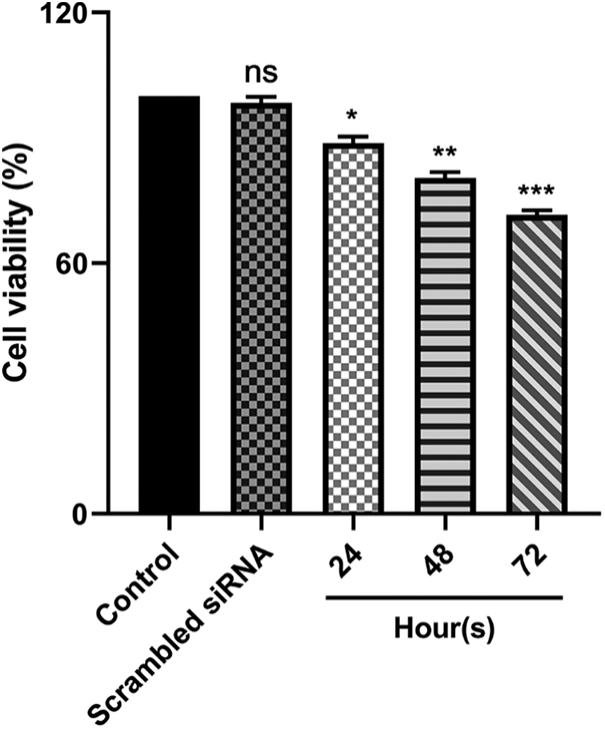
Effect of E5-siRNA on the cell viability in 24 h, 48 h, and 72 h after the transfection. 72 h after the transfection was the best time for cytotoxicity of siRNA (*p < 0.05, **p < 0.01, ***p < 0.001, n = 3).

Utilizing MTT assay, the cytotoxic effect of reduced E5 expression on CaSki cells was investigated 24, 48, and 72 h after transfection of cells with 100 picomole dose of siRNA. The findings revealed that cell transfections with E5-siRNA were 24, 48, and 72 h lower than control and scrambled siRNA-transfected groups (Fig. 2).

### The effect of E5-siRNA on apoptosis induction in CaSki cells

**Figure 3:**
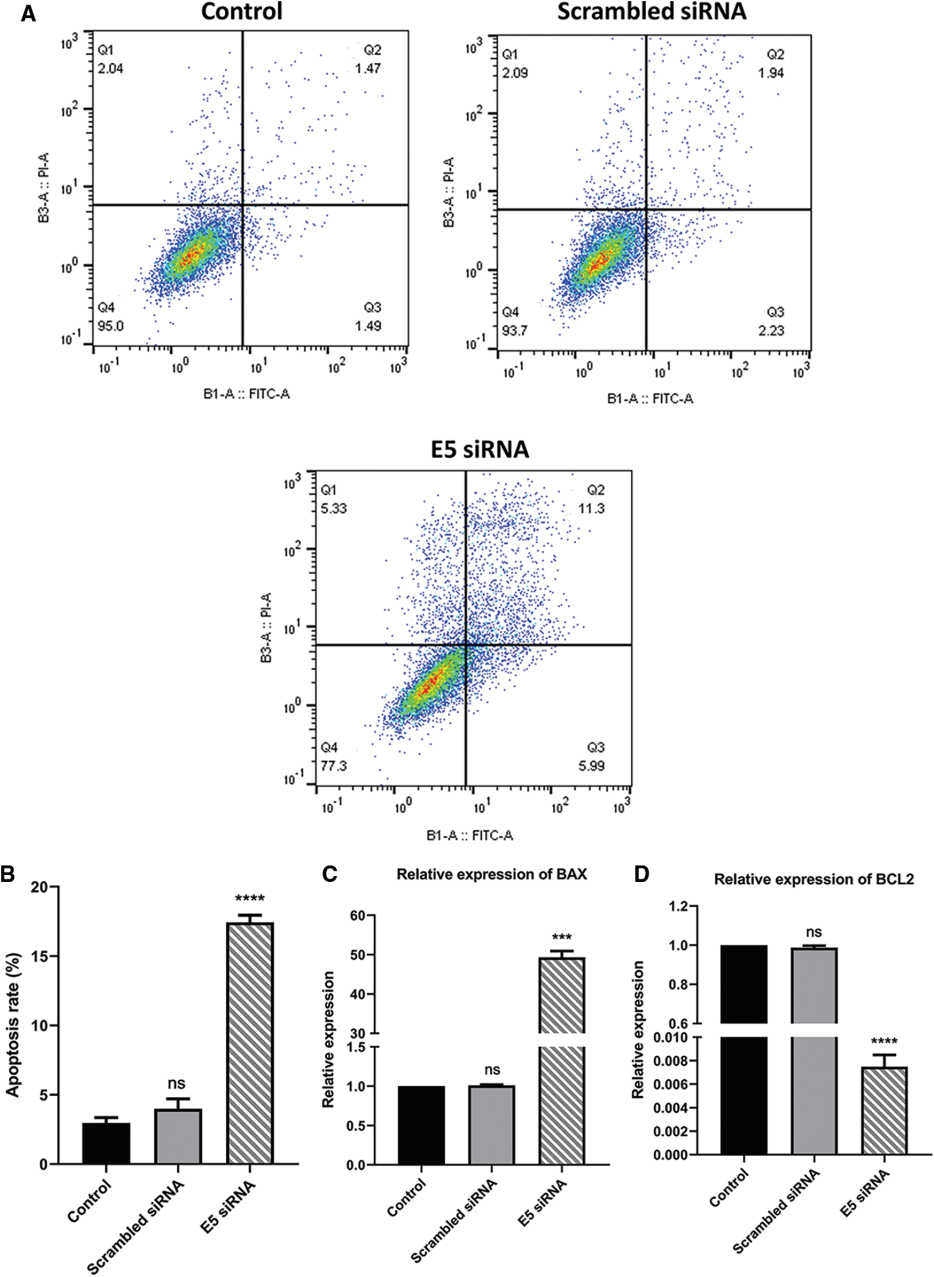
**A.** Annexin-V/PI assay for assessing apoptosis in control, scramble siRNA, and E5-siRNA group. **B.** The rate of apoptosis in the groups. The apoptosis rate significantly climbed in the test group compared with the control and scrambled siRNA-transfected group. **C.** Effect of E5-siRNA on the expression of *BAX*. *BAX* expression was upregulated after the transfection. **D.** Effect of E5-siRNA on the expression of *BCL2*. *BCL2* expression was downregulated after the transfection (***p < 0.001, ****p < 0.0001, n = 3).

To investigate the effect of E5-siRNA on apoptosis induction of CaSki cells, Annexin-V/PI staining was performed by flow cytometry. The results showed that apoptosis was significantly increased in cells transfected with siRNA compared to the control group. Also, this variable was not changed in cells transfected with scrambled siRNA (Figs. 3A and 3B). Thus, given the apoptosis rate in cells transfected with E5-siRNA slightly went up, the transfection of E5-siRNA might mostly reduce the antiapoptotic effect of E5 rather than induction of apoptosis. Moreover, *BAX* and *BCL2* expression was evaluated by qRT-PCR. A substantial rise in *BAX* expression and a striking decrease in *BCL2* expression level were seen after the transfection in the test group, which was in line with the apoptosis assay results (Figs. 3C and 3D).

### Effect of E5-siRNA on cell cycle process in CaSki cells

**Figure 4:**
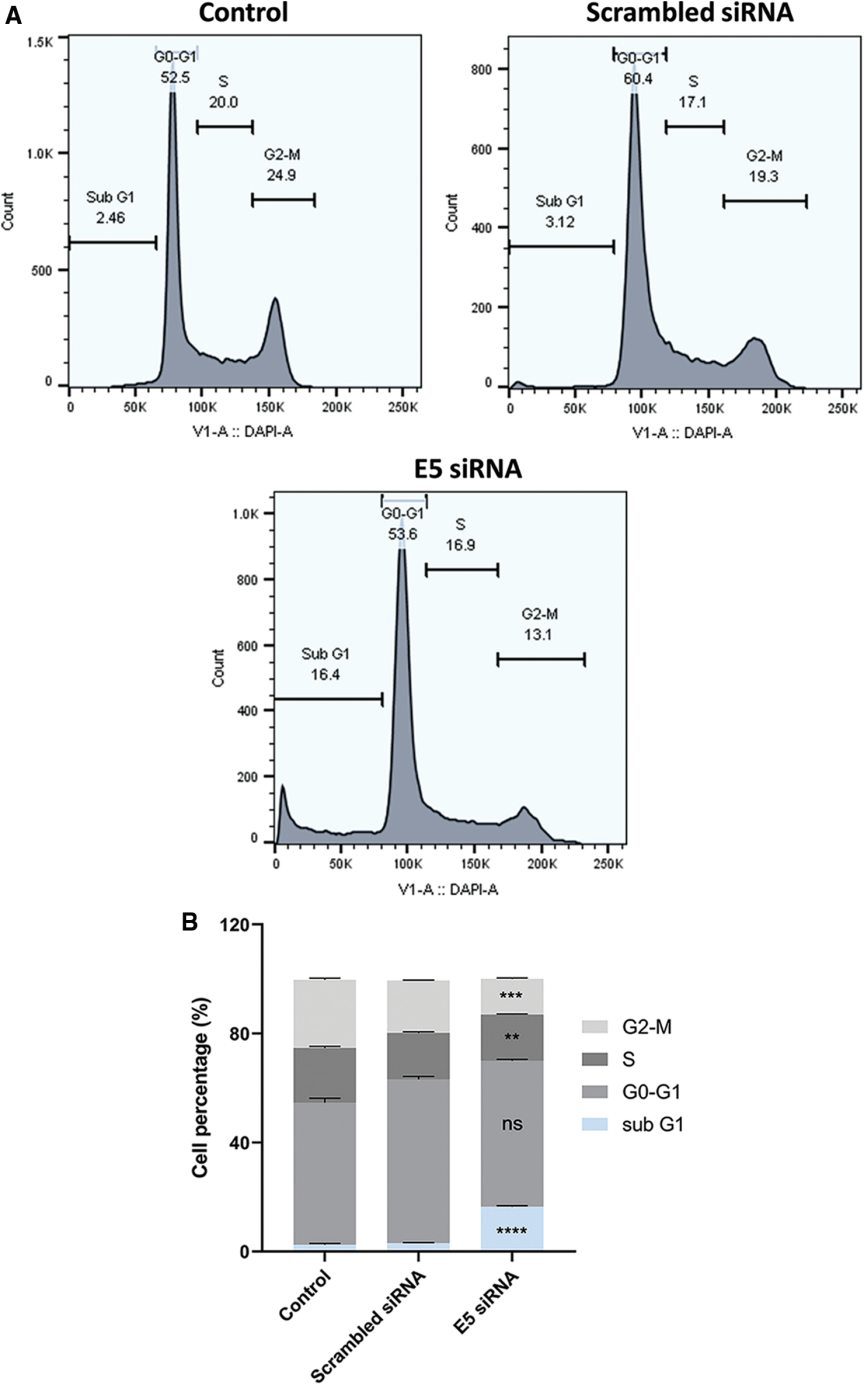
**A.** The assessment of the cell cycle by flow cytometry. The cell cycle arrest was shown in the test group. **B.** The different cell cycle arrests in groups. The test group cells were significantly arrested in sub-G1 (**p < 0.01, ***p < 0.001, ****p < 0.0001, n = 3).

Flow cytometry was used to investigate the effect of E5-siRNA on cell cycle arrest. According to the results, transfection of cells with E5-siRNA arrested the cell cycle progression at the sub-G1 phase (Fig. 4A). The percentage of cells stopped at the sub-G1 phase was 2.46% and 3.12% in control and scrambled siRNA-transfected groups, respectively, significantly different from the 16.4% of arrested cells in the E5-siRNA transfected group (Fig. 4B).

### E5-siRNA does not have any effect on the expression of EGFR; however, it could decrease the expression of PTGS2 (COX-2)

**Figure 5:**
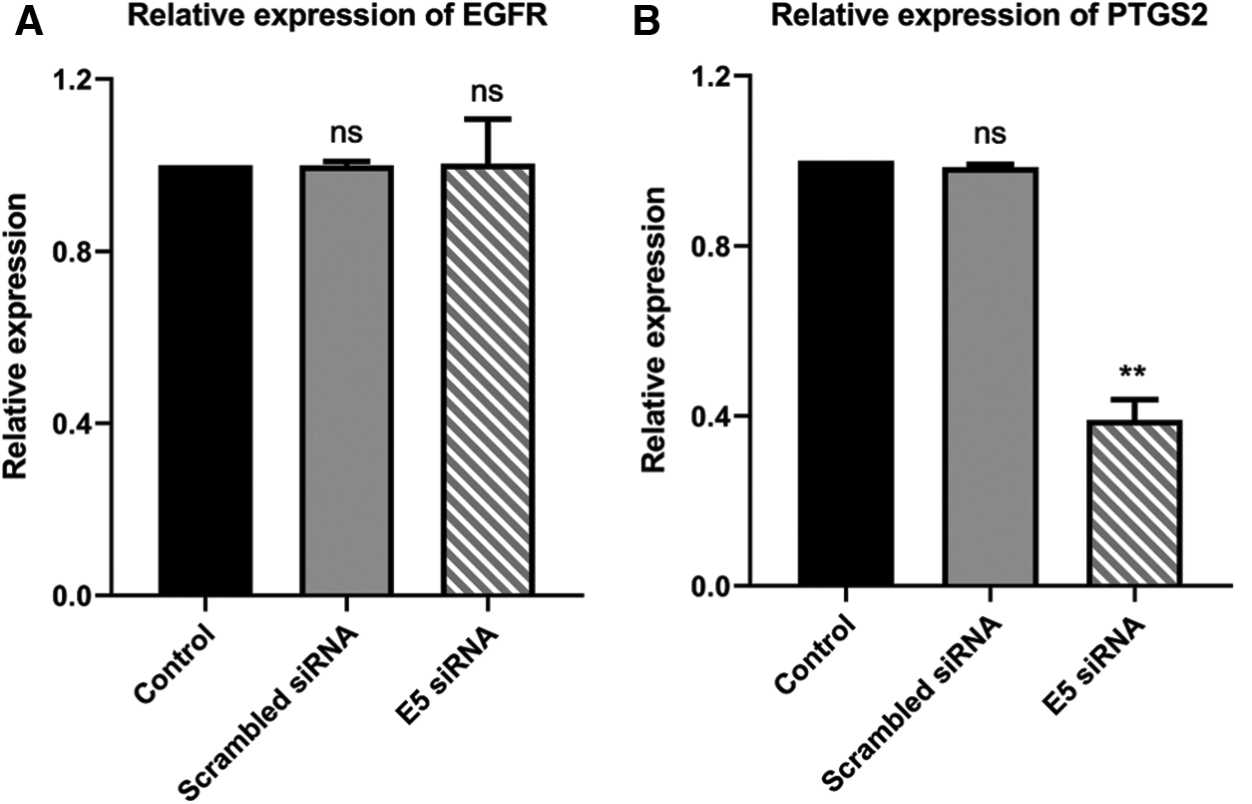
**A.** Effect of E5-siRNA on the expression of EGFR. The E5 suppression did not have any effect on EGFR expression. **B.** Effect of E5-siRNA on the expression of PTGS2. PTGS2 expression was downregulated after the transfection (**p < 0.01, n = 3).

The reduced level of E5 in HPV-16-positive cervical cancer cells showed no significant alteration in the *EGFR* expression level in the test group compared to the control and scrambled siRNA groups (Fig. 5A). However, there was a slight decrease in the *PTGS2* (COX-2) expression level in the test group (Fig. 5B), which in turn confirmed the ameliorated proliferation and inflammation in these cells.

### E5-siRNA could have an inhibitory effect on the migration of CaSki cells

**Figure 6:**
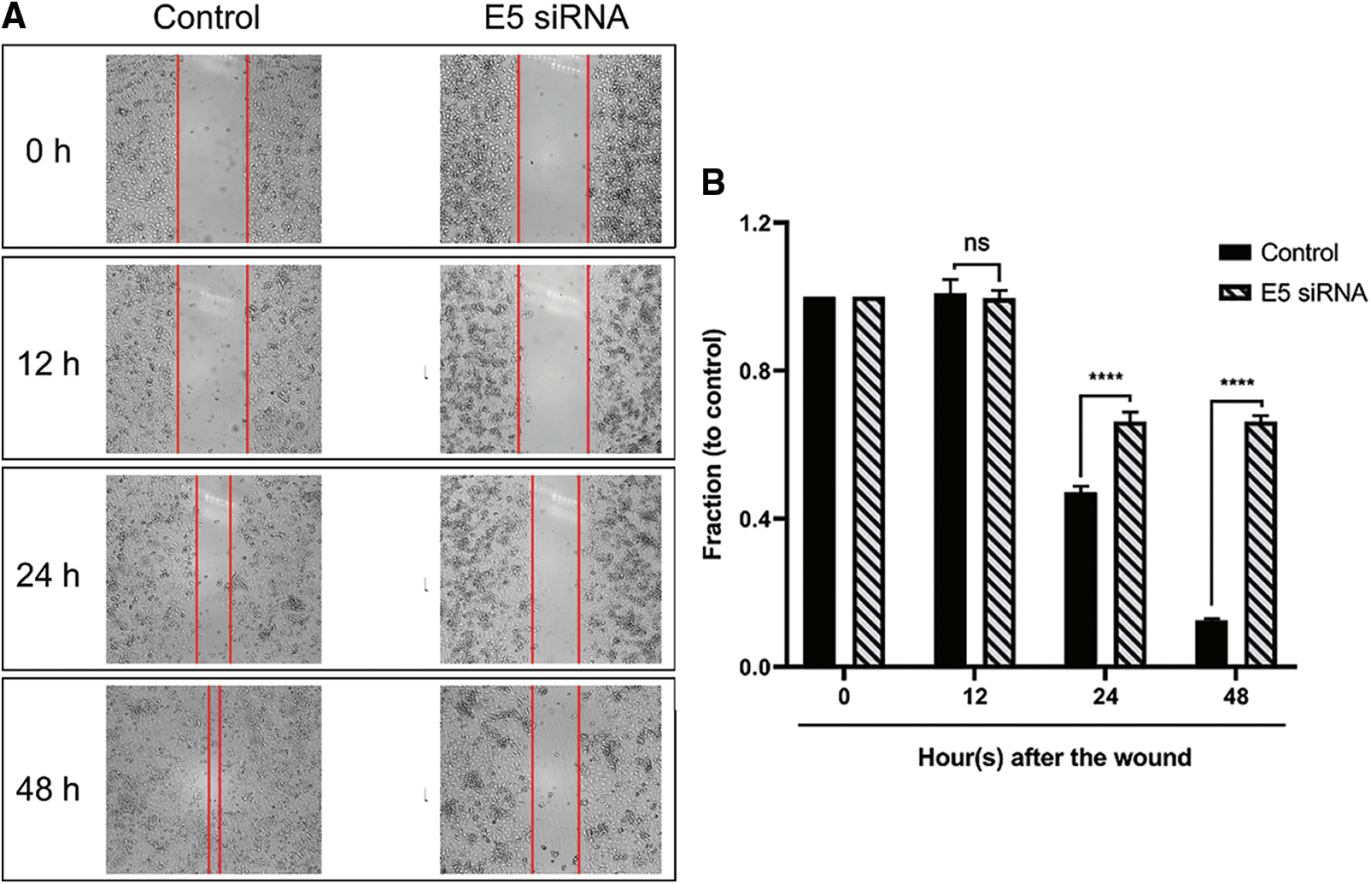
**A.** The results of the wound healing assay after E5-siRNA transfection. The time-dependent ability of E5-siRNA on the migration and motility rate of CaSki cells could be objectively significant. **B.** Transfection of E5-siRNA significantly decline the motility of CaSki cells. The results showed that E5-siRNA after 48 h upon the transfection have the most inhibitory ability on the migration of CaSki cells compared to the control group (****p < 0.0001, n = 3).

The wound-healing assay results (scratch) showed that the transfection of cervical cancer cells with E5-siRNA could objectively hamper the migration ability of these cells and decrease their motility (Figs. 6A and 6B).

## Discussion

Cervical cancer is one of the most prevalent cancers for women and is the third leading cause of annual death from women’s cancer. This cancer can have different reasons, but infection with some types of HPV, smoking, and HIV infection are essential factors in developing this malignancy [16]. Three early oncogenes (E5, E6, and E7) that affect cellular proliferation, differentiation, and survival are encoded by high-risk HPV. As the main drivers of keratinocyte proliferation, the functions of E6 and E7 are well defined, and their expression is maintained within tumors [17]. However, E5, which is revealed to have a pivotal effect on cellular and signaling pathways in human cell lines, is less acknowledged [18]. High-risk expression of E5 induces anchorage-independent development in murine fibroblasts and low serum growth and primary human keratinocyte conversion [19,20]. Therefore, E5 is a promising target for antiviral and anti-tumor-potential medicines. Unfortunately, despite their benefits, current chemotherapy treatments usually done for cervical cancer patients have disadvantages that reduce their efficacy and effectiveness. However, gene therapy is a powerful form of targeted therapy that interferes with expressing a particular gene in cells. In the present investigation, we examined the biological effect of HPV E5 oncoprotein in CaSki cells by suppressing this gene by E5-siRNA.

The current study investigated the cytotoxic effects of E5 suppression on cell survival and cell proliferation using an MTT assay. The results of the MTT assay showed that the transfection of cells with E5 siRNA decreased the survival and proliferation of transfected cancer cells compared to the control group, and this decrease was completely time-dependent. Furthermore, Liao et al. have also shown that HPV 16/18 E5 can increase the proliferation of cervical cancer cells, which approves our finding of the anti-proliferative effect of E5-siRNA [21].

In addition, the results of Annexin-V/PI staining were similar to those expected with MTT results and showed that E5 siRNA significantly induces apoptosis in cervical cancer cells compared to the control group. A comparison of MTT and Annexin-V/PI results showed that most of the reported cell death in MTT was due to apoptosis induction. Oh et al. [22] also demonstrated that HPV E5 could suppress the apoptosis and dysregulation of apoptosis-related gene expression; the knockdown of E5 by siRNA elevated the apoptosis rate. When reactive oxygen species (ROS)-induced apoptosis occurs, HPV16 E5 promotes the proteasomal degradation of the pro-apoptotic Bcl-2 family member BAX. Moreover, by suppressing Fas receptor expression and preventing recruitment of Fas-associated protein with death domain (FADD) to form the death-induced signaling complex (DISC), HPV16 E5 can reduce FasL-and TNF-related apoptosis-inducing ligand (TRAIL)-induced apoptosis. E5-dependent stimulation of EGFR and downstream signaling via phosphatidylinositol 3-kinase (PI3 K) and extracellular signal-regulated kinase 1/2 (ERK1/2) are required for HPV16 E5 to decrease the apoptotic response to ultraviolet (UV)-B radiation [23]. In the current study, the results of qRT-PCR showed that suppression of E5 could significantly affect the apoptosis rate by modulating the expression of BAX and BCL2.

The results of cell cycle analysis confirmed the previous works that transfection of cells with siRNA E5 in the cell cycle pattern caused significant changes and induced cell cycle arrest at the sub G1 phase compared to the control group. These results may indicate the anti-proliferative and inhibitory effect of siRNA E5 on cell cycle patterns. Furthermore, it has been demonstrated that the HPV E5 oncoprotein might affect the cell cycle and progress the HPV-infected cells initiating the cell cycle [24].

Regarding the involvement of the EGFR signaling pathway in the induction and progression of cervical cancer and the ability of E5 to increase the EGFR recycling process within the HPV-infected cells, it is recommended that the E5 protein could exacerbate the severity of cervical cancer via elevating the activity of EGFR signaling pathway [25]. To prove the relation between E5 and EGFR signaling pathway, the suppression of E5 was done. The results demonstrated that the inhibition of E5 expression does not affect the expression of EGFR. In 2019, Basto et al. [26] reported that for HPV16 + and HPV18 + cancers, there was a link between the presence of E5 transcripts and viral genome disruption. No correlation was found between tumors capable of translating E5 and EGFR or VEGFA transcriptional levels. However, COX2 (PTGS2), as one of the most critical downstream genes of EGFR, was significantly decreased. Jung-Min Oh et al. also showed that the suppression of E5 could reduce the expression of COX2 in CaSki cells. COX2 ability to induce inflammation during HPV infection highlights another function of E5 to trigger inflammation, which should be investigated by further studies [27].

In conclusion, this study revealed that the suppression of HPV-16 E5 oncogene could have an inhibitory effect on the proliferation and triggering apoptosis in HPV-16-positive cervical cancer cells, which suggests that suppression of HPV-16 oncogenes, in particular E5, could significantly affect the progression of cervical cancer. However, several aspects of this therapeutic approach still remain unclear and need to be investigated by further *in-vivo* and human studies.

## Data Availability

All data are presented in the article and can be accessed by communicating with the corresponding author.
